# The Implication of the Brain Insulin Receptor in Late Onset Alzheimer’s Disease Dementia

**DOI:** 10.3390/ph11010011

**Published:** 2018-01-29

**Authors:** Jaume Folch, Miren Ettcheto, Oriol Busquets, Elena Sánchez-López, Rubén D. Castro-Torres, Ester Verdaguer, Patricia R. Manzine, Saghar Rabiei Poor, María Luisa García, Jordi Olloquequi, Carlos Beas-Zarate, Carme Auladell, Antoni Camins

**Affiliations:** 1 Departament de Bioquímica i Biotecnologia, Facultat de Medicina i Ciències de la Salut, Universitat Rovira i Virgili, 43201 Reus, Spain; jaume.folch@urv.cat (J.F.); emiren@gmail.com (M.E.); oriolbusquets@gmail.com (O.B.); 2 Biomedical Research Networking Centre in Neurodegenerative Diseases (CIBERNED), 28031 Madrid, Spain; esanchezlopez@ub.edu (E.S.-L.); rubendario@gmail.com (R.D.C.-T.); everdaguer@ub.edu (E.V.); cauladell@ub.edu (C.A.); 3 Departament de Farmacologia, Toxicologia i Química Terapèutica, Facultat de Farmàcia i Ciències de l’Alimentació, Universitat de Barcelona, Av. Joan XXIII 27/31, E-08028 Barcelona, Spain; patricia_manzine@yahoo.com.br (P.R.M.); shagar.rabii@gmail.com (S.R.P.); 4 Institut de Neurociències, Universitat de Barcelona, E-08028 Barcelona, Spain; 5 Unitat de Farmàcia, Tecnologia Farmacèutica i Fisico-química, Facultat de Farmàcia i Ciències de l’Alimentació, Universitat de Barcelona, E-08028 Barcelona, Spain; rdcm@ub.edu; 6 Institute of Nanoscience and Nanotechnology (IN2UB), University of Barcelona, Barcelona E-08028, Spain; 7 Departament de Biologia Cel·lular, Fisiologia i Immunologia, Facultat de Biologia, Universitat de Barcelona, E-08028 Barcelona, Spain; 8 Laboratorio de Regeneración y Desarrollo Neural, Instituto de Neurobiología, Departamento de Biología Celular y Molecular, Centro Universitario de Ciencias Biológicas y Agropecuarias, Universidad de Guadalajara, Zapopan 44600, Mexico; carlosbeas55@gmail.com; 9 Department of Gerontology, Federal University of São Carlos (UFSCar), São Carlos 13565-905, Brazil; 10 Instituto de Ciencias Biomédicas, Facultad de Ciencias de la Salud, Universidad Autónoma de Chile, Talca 3460000, Chile; jolloquequi@gmail.com

**Keywords:** Alzheimer’s, insulin resistance, amyloid, TAU, cognition, insulin receptor, type 2 diabetes

## Abstract

Alzheimer’s disease (AD) is progressive neurodegenerative disorder characterized by brain accumulation of the amyloid β peptide (Aβ), which form senile plaques, neurofibrillary tangles (NFT) and, eventually, neurodegeneration and cognitive impairment. Interestingly, epidemiological studies have described a relationship between type 2 diabetes mellitus (T2DM) and this pathology, being one of the risk factors for the development of AD pathogenesis. Information as it is, it would point out that, impairment in insulin signalling and glucose metabolism, in central as well as peripheral systems, would be one of the reasons for the cognitive decline. Brain insulin resistance, also known as Type 3 diabetes, leads to the increase of Aβ production and TAU phosphorylation, mitochondrial dysfunction, oxidative stress, protein misfolding, and cognitive impairment, which are all hallmarks of AD. Moreover, given the complexity of interlocking mechanisms found in late onset AD (LOAD) pathogenesis, more data is being obtained. Recent evidence showed that Aβ42 generated in the brain would impact negatively on the hypothalamus, accelerating the “peripheral” symptomatology of AD. In this situation, Aβ42 production would induce hypothalamic dysfunction that would favour peripheral hyperglycaemia due to down regulation of the liver insulin receptor. The objective of this review is to discuss the existing evidence supporting the concept that brain insulin resistance and altered glucose metabolism play an important role in pathogenesis of LOAD. Furthermore, we discuss AD treatment approaches targeting insulin signalling using anti-diabetic drugs and mTOR inhibitors.

## 1. Introduction

Prevention is a key factor when trying to reduce the impact of age-related diseases like cardiovascular alterations, cancer and dementias. These pathologies do not only create substantial personal and family burdens but, unsustainable increases in the public health economic costs in developed populations. The most common form of dementia is Alzheimer’s disease (AD) [1,2,3,4,5,6,7]. The number of patients diagnosed with AD is rapidly increasing worldwide and becoming a common cause of death in aging populations [4,5,6,7]. Moreover, no effective treatments have been established yet to prevent or delay the progression of AD [8,9]. 

AD has been classified into two groups, depending on its onset: the first classification is familial AD, related to genetic alterations of the amyloid beta precursor protein (AβPP) and preselinins (PS1) [1,2,3,4,5]. This subgroup represents approximately about 3% of the diseased patients [1,4]. The other classification group is the late onset form (LOAD), also known as sporadic. It accounts for the remaining 97% of diagnoses [1,4]. Historically, the neuropathological characteristics of AD were described through the amyloidogenic hypothesis by Selkoe (1992) [9,10]. They were: cognitive loss, abnormal accumulations of Aβ and hyperphosphorylation of TAU protein in areas of the cerebral cortex and hippocampus [6,7,8,9,10]. Initially, the Aβ peptide is generated from the catabolism of AβPP, a plasmatic membrane protein with a single domain found in different cellular types, including neurons, astrocytes and oligodendrocytes [10,11]. This protein is cleaved by α-, β-, and γ-secretase enzymes, as well as, a complex of proteins containing presenilin 1 (PS1) [4,10,11]. In neuropathological situations, AβPP is metabolized predominantly by the amyloidogenic pathway in which the Aβ cleaving enzyme 1 (BACE 1; β-secretase) breaks AβPP by the N-terminal end while γ-secretases cleave the C-terminal end, obtaining Aβ40 and Aβ42 fragments that remain in the extracellular space [4,10].

Since the presence of amyloid plaques has been repeatedly demonstrated not to be strictly correlated with AD symptoms, considerable research has focused on understanding its actual role. Now, one of the working hypotheses is that Aβ42 accumulates in the form of several soluble species, oligomers and protofibres, which contain potentially high toxic properties [11]. As the concentration of these molecules increases, they continue to aggregate into insoluble fibres that are the main constituents of Aβ plaques. Nevertheless, some authors suggest that the cognitive impairment correlates best with alterations on the TAU protein than the Aβ plaques, suggesting a predominant role for TAU in the pathogenesis of AD [4,9,10,11]. Therefore, in the field of AD research, there are multiple approaches to consider when trying to understand the origin of this disease.

Another hallmark that has been associated as a risk factor for LOAD is the ε4 allele of the apolipoprotein E (APOE) gene [12,13,14,15,16]. This information was demonstrated when studying how the response to intranasal insulin differed between carriers of different apolipoprotein ε4 alleles [14]. APOE genotype influences peripheral glucose and insulin metabolism. Also, as it occurs in other diseases, gender has an influence in the affection and incidence of pathology. APOE positive carrier women have higher risk than men to develop LOAD and, respond less favourably to insulin related therapies [13,14,15,16]. Importantly, the molecular basis of this association has remained elusive for decades. Yet, recent findings determined that APOE4 interacts with insulin receptors (IR), impairing its trafficking between endosomes and the plasmatic membranes, by trapping them and favouring the development of impaired insulin signalling [16]. Furthermore, the association between T2DM and LOAD amyloid pathology is specific among carriers of the apolipoprotein E (APOE) ε4 gene allele, compared to the common ε3 allele and the protective ε2 allele [16]. All these new data reinforce the metabolic hypothesis that T2DM appears as a key factor involved in LOAD [17,18,19,20].

Currently, only symptomatic therapies are available and their effects are modest (acetylcholinesterase inhibitors and NMDA antagonists) [8]. Memantine (MEM), a low-affinity voltage-dependent uncompetitive antagonist of NMDA receptors (NMDAR), is currently being prescribed for the treatment of AD, jointly with acetylcholinesterase inhibitors such as galantamine, donepezil, and rivastigmine [20,21,22]. Since it is a low-affinity antagonist, it blocks the NMDAR but it is rapidly displaced from it, avoiding prolonged receptor blockade and the associated negative side effects on learning and memory that have been observed in high affinity NMDAR antagonists. Unfortunately, and despite its high prevalence and mortality, there are no effective disease-modifying therapies at present.

For the last 25 years, the main focus of research has been on senile plaques, considering them the main source of the symptoms of AD. Consequently, therapeutic approaches have focused on this biomarker. Recent studies at preclinical level and in LOAD patients show that an antibody (aducanumab) penetrates in the brain and reduces soluble and insoluble Aβ42 in a dose-dependent manner [23]. In patients with mild LOAD, a year of treatment by administering monthly intravenous infusions of aducanumab, reduces cerebral Aβ and patients show cognitive improvements. These results suggest that the amyloidogenic hypothesis may contribute to the development of LOAD exacerbating its consequences, possibly along with other factors such as metabolic alterations, glia activation, mitochondrial alteration and oxidative stress, among others [23]. Thus, the classical definition of AD that attributes the main role to plaques and tangles as the main responsible source of the neuropathophysiology should be modified. 

Accordingly, the main objective of this review is to summarize the information regarding metabolic alterations and the appearance of LOAD, especially associated with type II diabetes mellitus (T2DM). Finally, the pathways associated with the IR signalling and its inhibition will be presented. Our aim is to evaluate the possible application of drugs involved in the regulation/modulation of brain IR in LOAD treatment, in order to improve cognitive performance and deter the development of AD.

## 2. The Hippocampal Insulin Receptor Is a Key Target in Physiological Cognitive Processes and Neurodegeneration 

In 1985, previous to the establishment of the amyloidogenic hypothesis as the paradigm for the study of AD, Hoyer, proposed the concept of central insulin resistance and dysfunctional insulin signalling in LOAD (Table 1) [24,25,26,27,28]. Insulin resistance is defined as a situation in the human organism in which it does not respond sufficiently to the physiological levels of insulin [29,30,31,32,33,34,35,36,37,38,39]. It is involved in the onset of the metabolic syndrome. Yet, even though this idea had already been theorized, these conclusions did not begin to be established until the publication of the Rotterdam study, a clinical report that described the connection between T2DM and LOAD, revealing that those patients that had been diagnosed with diabetes had higher risk of dementia [33,34]. Subsequent clinical and epidemiological studies have confirmed this potential association demonstrating that the alteration of metabolic parameters, such as hyperglycaemia and hyperinsulinemia, correlates positively with the development of pathology related to LOAD, mainly with the cognitive loss [35,36,37,38,39,40,41].

**Figure 1:**
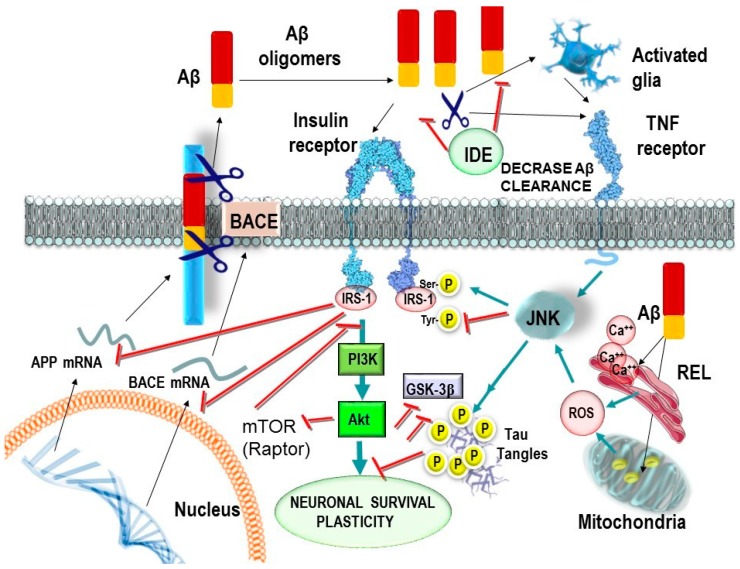
Consequences of insulin and Aβ interactions on reduced neuronal IR signalling. In type 2 diabetes, there can be decreased or increased levels of insulin in brain, along with IR desensitization. Soluble Aβ oligomers block IR. Increased Aβ levels will compete for insulin degrading enzyme (IDE) against cerebral insulin. Reduced IR signalling results in downstream negative effects on PI3K activity and proteins like PKB/AKT. Consequences of this include: reduced glucose metabolism and increased oxidative stress which modulate APP and JNK activity. Moreover, reduced GSK3β phosphorylation leads to up-regulation in TAU phosphorylation and Aβ formation. Likewise, Aβ promotes the activation of microglia increasing the levels of cytokines, mainly TNFα that activates JNK that subsequently inhibits the brain IR. On the other hand, Aβ can alter the endoplasmic reticulum and the mitochondria, generating free oxygen radicals that modulate APP and JNK.

Under physiological conditions when insulin binds to the IR, a cascade regulates key downstream serine/threonine kinases such as, protein kinases B (AKT/PKB), mechanistic target of rapamycin (mTOR), and extracellular signal-regulated kinases (ERK), that eventually phosphorylate serine/threonine residues of the insulin receptor substrates (IRS), inhibiting insulin signalling in a negative feedback regulation (Figure 1) [35,36,37,38,39,40,41,44,45,46]. In neurons, the phosphoinositide 3-kinase (PI3K), AKT, glycogen synthase kinase 3β (GSK3β), BCL-2 agonist of cell death (BAD), fork-head box (FOX), mTOR and the mitogen activated protein kinase (MAPK) pathways are critical for cell survival signalling and are regulated by the activity of the IR [43,47,48]. Therefore, alteration of the physiological activity on these pathways might be the source of alteration in normal neuronal performance, supporting the hypothesis that brain insulin resistance could promote LOAD, precisely by inhibition of these pathways [39,41,45].

The metabolic hypothesis associated with the appearance of LOAD is based on the fact that cerebral IRs are widely distributed in the brain, existing in higher densities in the olfactory bulb, hypothalamus, cerebral cortex and hippocampus [28,49]. Frölich and colleagues reported a significant reduced level of CNS IRs in LOAD patients [24,25,26,27,28]. Research from the group led by de la Monte, demonstrated significant decreases in insulin and insulin growth factor (IGF-I) receptor levels in LOAD frontal cortex, hippocampus, and hypothalamus of AD patients [45,50]. In addition, the same research group correlated the decrease in gene expression and protein levels of insulin, IGF1 receptors and other downstream molecules, with impaired acetylcholine production and cognitive performance in LOAD brains. Another recent study strengthened this hypothesis by demonstrating significant alterations in mRNA expression profiles of genes related to insulin signalling in the cortex and hippocampus [51]. Intriguingly, the highest differences in mRNA expression were detected in the hippocampal region of the brain, the main area associated with the cognitive process [51]. 

A recently published work by the group of Butterfield, reported new information about the complexity of LOAD [44]. In essence, they suggested that human and preclinical studies have provided convincing evidence that in the brains of many LOAD patients and rodents there is a decrease in energy metabolism and, in particular, a decrease in glucose utilization. As a consequence, that LOAD will represent a metabolic disease in which brain glucose utilization and energy production are altered is gaining attention [24,25,26,27,28,39]. Additional data by Grillo and co-workers reported in a very interesting study, that insulin resistance in the hippocampus would prevent the correct structuring of memory, which would be directly related to cognitive loss [42,52]. The administration of a lentiviral vector expressing an antisense sequence of the brain IRs to rats caused for cognitive loss. Using this experimental approach, the authors were able to decrease the number of IR in the hippocampus without affecting peripheral glucose homeostasis, thus generating a specific rat model of altered brain insulin signalling in the hippocampus [42]. This study demonstrated that insulin resistance in the hippocampus might induce a neuroplasticity deficit, including deficits in spatial learning and memory. In addition, the hippocampal levels of the phosphorylated GluN2B and GluA1 subunits were reduced, providing a possible molecular evidence on how the deficit in synaptic transmission occurs when there are alterations on the insulin signalling in the hippocampus [52]. 

Concurrently, the de Felice research group has demonstrated that Aβ oligomers bind to IR, causing for their removal from the neuronal surface membrane, causing its cellular internalization and, thereby providing an evidence for brain insulin resistance in LOAD [19,29]. In addition, some authors have reported that insulin prevents detrimental effects of Aβ oligomers on the inactivation of brain IR [53]. Furthermore, insulin promotes the release of intracellular synaptic Aβ, and regulates expression of insulin-degrading enzyme (IDE), a protease involved in clearance of Aβ (Table 1) [17].

A possible conclusion from the outcome of these studies might suggest that the presence of Aβ peptide may not be the only factor necessary for cognitive loss. However, Aβ paired with a process of obesity and hence, peripheral and central insulin resistance, may exacerbate the onset of LOAD and worsen cognitive loss. Then, LOAD should be considered globally as a brain expression of a metabolic disease of the whole organism, and correspondingly should not focus only on events that occur in the brain [32].

## 3. Molecular Bases of Insulin Receptor Modulation

Insulin is a peptide hormone of 5.8 KDa that is synthesized and secreted by the pancreatic β cells. Once released to the blood vessels, insulin is transported to the brain through the blood brain barrier (BBB) and binds to their cognate receptors [24,25]. The IR is a glycoprotein consisting of an extracellular α subunit (135 KDa) that inhibits the activity of the β-transmembrane subunit (95 KDa) [26,27,28]. The IRs belongs to the tyrosine kinase receptor superfamily. When insulin binds to the α subunit, it dimerizes to form the α2β2 complex in the cell membrane, it leads to autophosphorylation of the beta subunit on Tyr1158, Tyr1162, and Tyr1163, which constitutes the first step in IR activation. 

It has been shown that the activation of the IR tyrosine kinase, leads to the recruitment and phosphorylation of several substrates, including IRS1-4, the adapter protein SHC, growth factor receptor-bound protein-2 (Grb-2 or GAB1), dedicator of cytokinesis (DOCK1), casitas B-lineage lymphoma (CBL) and an interacting protein called APS which are associated proteins, all of which provide specific binding sites for the recruitment of other proteins of the signalling cascade [49]. These phospho-tyrosine residues bind to IRS 1 and 2 in order to initiate several signalling pathways, including the PI3K-AKT pathway [54,55]. PI3K converts phosphatidylinositol-4,5-bisphosphate (PIP2) to phosphatidylinositol-3,4,5-trisphosphate (PIP3). This conversion favours the activation of the PKB/AKT through the 3-phosphoinositide dependent kinase (PDK). PIP3 recruits AKT to the plasma membrane, where it becomes phosphorylated by 3-phosphoinositide-dependent protein kinase 1 (PDK1), which regulates the translocation of glucose transporter type 4 (GLUT4) to the plasma membrane in the hippocampus.

In the brain, IR activation promotes neuronal survival through the phosphorylation of the FOXO transcription factor, favouring its way out of the nucleus of the cell [55,56,57,58]. FOXO is a transcription factor involved in the expression of pro-apoptotic mediators, thus contributing to the process of cell death. All this processes that are regulated by the signalling of the IR, result in deleterious effects on synaptic function and cognitive impairment. The activation of IR tyrosine kinase also results in the activation of the RAS/MAPKs pathway. The stress activated protein kinases (SAPK) or MAPK, include extracellular signal-regulated kinases 1 and 2 (ERK1 and ERK2), p38 and the c-Jun-N-terminal kinases (JNKs) [56]. The JNK family is made up by 3 genes that codify for 10 different products classified into 3 different isoforms: JNK1 (*Mapk8*), JNK2 (*Mapk9*) and JNK3 (*Mapk10*) and while JNK1 and JNK2 are ubiquitously expressed, JNK3 expression is principally restricted to regions of the brain, heart and testis. When activated, the JNK1 phosphorylates IRS-1 in the serine residues (IRS-1pSer) [29]. This alteration blocks the signalling of the insulin pathway and favours peripheral resistance to this hormone. Based on this activation sequence, JNK1 appears as a key protein to investigate novel therapeutic targets that prevent the development T2DM [54,56,58].

## 4. Relationship between Insulin Receptor Activation and TAU Phosphorylation

Recent studies have suggested a potential link between impaired insulin signalling and pathogenic alterations of TAU. As we stated before, the activation of IR, IRS1 and 2 initiates several signalling pathways, including the conversion of PIP2 into PIP3, which favours the activation of PKB/AKT and, consequently, leads to the translocation of GLUT4 to the plasma membrane [56,59,60,61,62]. Concurrently, AKT signalling affects other diverse cellular responses, like neuronal survival and TAU phosphorylation. Other important targets such as the GSK3β are also regulated by AKT, inhibiting its activation by phosphorylation. Insulin resistance reinforces the activation of GSK3β leading to increased phosphorylation levels of TAU protein and, the subsequent formation of neurofibrillary tangles, one of the hallmarks of AD neuropathology [59,60,61,62]. Schubert and co-workers investigate the biochemical processes associated with neurodegeneration in a brain specific IR knockout (NIRKO) mice model [63]. They reported that NIRKO mice presented decreased AKT activity thus, having the previously mentioned increases in GSK3β activation and TAU hyperphosphorylation at specific sites associated with LOAD. This data, along with other being produced in the same line on the study of metabolic alterations, confirm how altered insulin signalling in the brain leads to the appearance of classical hallmarks of LOAD and, demonstrates, how neuronal insulin resistance predisposes for the appearance of pathologies. Freude and co-workers reported that brain insulin receptor specifically modulates TAU phosphorylation at Ser202, a key site which predisposes for tangle formation after peripheral insulin injection in mice [64]. The effects of injected insulin on TAU were abolished in the NIRKO mice.

Studies performed in *post-mortem* brains from patients with tauopathies including AD, Pick’s disease, corticobasal degeneration, and progressive supranuclear palsy, showed increases in phosphorylated IRS1 levels which, as we have already mentioned, is a specific marker of insulin resistance. Interestingly, two independent research groups published their research on alterations in brain insulin receptors and downstream pathway in LOAD. Liu and colleagues reported that the insulin-signalling pathway is decreased in LOAD brain and demonstrated that alteration in insulin signaling may contribute to LOAD through the hyperphosphorylation of TAU [59]. In addition, authors suggest that brain insulin resistance is also correlated with calpain activation, a protease involved in cyclin-dependent kinase (CDK5) activation, a kinase involved in the phosphorylation of TAU [35]. Similar results were reported by other authors:

- Talbot and co-workers reported that LOAD patients show impaired brain insulin-signaling transduction with reduced tyrosine kinase activity of the IR [35]. IR and its receptor analogous IGF1R form heterodimers (IR/IGF1R) that modulate the selectivity and affinity for insulin and IGF1 in the activation of signaling molecules [65].
- Yarchoan and co-workers reported an increase in serine phosphorylation of IRS1 (inactivation), the phosphorylation of IRS1 on multiple serine residues can inhibit IRS1 activity, leading to insulin resistance in the hippocampus in LOAD and other tauopathies [54].

Finally, recent data reported that insulin accumulates intraneuronally together with hyperphosphorylated TAU in LOAD and several other tauopathies suggesting that hyperphosphorylated TAU-bearing neurons is a causative factor involved in the brain insulin resistance observed in LOAD (Table 1) [66]. 

## 5. Role of the Glucose Transporter 4 in Cognition

Glucose transporter 4 (GLUT4) is found in peripheral tissues like the skeletal muscle, heart, and adipose tissue [67,68,69]. Its role in the physiology of the cell is mainly the transport from the extracellular space into the citosol for its metabolism. Thus, in response to the activation of the IR signaling cascade by insulin, GLUT4 is translocated to the plasma membrane to facilitate glucose entry into the cell. Moreover, GLUT4 is found in brain regions such as the cerebellum, and especially the hippocampus. At the hippocampal level, the cognitive improvement effects related to insulin may occur via upregulation in GLUT4-mediated glucose uptake [68]. Thus hippocampal GLUT4 overexpression could be a target to improve the cognitive process in AD. This could be the case of quercetin which improves cognitive dysfunction mediated by chronic unpredicted stress, through upregulation of GLUT4 expression in the hippocampus [69].

## 6. Effects of Aβ Oligomers on Brain Insulin and Peripheral Metabolic Tissues 

Recent hypothesis suggests that since diabetes increases both Aβ production and TAU phosphorylation, both T2DM and Aβ may cooperate to induce neurodegeneration in LOAD [70,71]. It has been pointed out that soluble Aβ peptide oligomers would act as synaptotoxins [10,11,12]. Moreover, since Aβ and insulin are both amyloidogenic peptides sharing a common sequence recognition motif, it is possible that both molecules are able to bind to the IR. Given this assumption, Aβ may also potentiate insulin resistance through antagonistic effects, blocking the downstream pathway and facilitating the phosphorylation of GSK3β. Thus, the aging process associated with insulin resistance, jointly with Aβ production and hyperphosphorylation of TAU, can have a synergic effect leading to neuronal dysfunction. 

In addition to the effects of Aβ oligomers on TAU phosphorylation, a recent study using a mice model of AD, reported peripheral metabolic changes in plasma and liver extracts [72]. Also, Zhang and co-workers demonstrated in APPswe/PS1E9 mice that the Aβ42 peptide induces hepatic insulin resistance in vivo through the activation of the Janus Kinase 2 (JAK2), suggesting that inhibition of Aβ42 peptide production in the brain may be a novel strategy for the treatment of insulin resistance and therefore T2DM [73,74,75]. 

As, an overview, we can state that LOAD has a multifactorial component and should be addressed as a disease affecting the whole organism [70,71]. Moreover, preclinical studies in rodents have established that the oligomers of Aβ administered directly to hippocampal neurons induce synaptic loss and neuronal dysfunction, which eventually leads to memory loss [11]. Likewise, intracerebroventricular (icv) administration of Aβ oligomers causes behavioral changes and AD-like pathology in primates, providing an excellent model for investigating AD-related mechanisms [72]. Furthermore, Clarke and co-workers reported that intracerebral injected Aβ causes peripheral glucose intolerance and insulin resistance, as well as, inflammatory processes in the hypothalamus and adipose tissue, along with alterations of GLUT-4 insulin-induced cell membrane translocation in skeletal muscle [76]. 

Accordingly, Aβ peptides generated in the brain reach the hypothalamus and alter the body’s energy balance, favouring the apparition of a T2DM. In this line, Arietta-Cruz et al. demonstrated an increase in plasma glucose levels when injecting β25-35 into the hypothalamus of rat as a consequence of enhanced hepatic glucose production [77,78]. 

In addition, generated brain Aβ could accumulate in peripheral tissues such as the pancreas and skeletal muscle contributing to the negative effect on peripheral glucose metabolism [79]. Thus, when trying to explain this complicated bidirectional process between LOAD and T2DM, recent reported data suggests the existence of something called Factor X, a molecule or pathway that would be the bridge between Aβ as responsible of LOAD and T2DM. In addition, authors suggest that characterization of Factor X will be important in order to the development of a potential therapeutic target for LOAD prevention [70].

## 7. Is BACE1 a Potential Bridge between Aβ and T2DM?

BACE1 is involved in LOAD as the enzyme responsible for the rate-limiting step in Aβ production through the cleaving of the amyloid precursor protein (APP). It has been demonstrated that monomers of Aβ1-42 augment BACE1 gene transcription activation through the MAPK8/JNK1-MAPK9/JNK2 signalling pathway and by interfering with its lysosomal degradation leading to an amyloid vicious cycle [80,81,82]. 

Interestingly, recent data demonstrated at the preclinical level that neuronal expression of human BACE1 causes systemic diabetic complications via the induction of hypothalamic impairment, insulin resistance, hepatic deficits and global glucose alterations [79,80]. Using the PLB4 mouse it was demonstrated that the risk of diabetes when BACE1 is overexpressed in neurons increases, providing for a complex mechanistic interaction between T2DM and LOAD. Human (h) BACE1 neurogenic knockout has recently been shown to induce Aβ accumulation, promotes brain inflammation and generates LOAD-like phenotypes in mice in the absence of expression of mutant APP, suggesting that BACE1 represents a molecular risk factor for AD related to the aging process [83]. 

Plucińskaí and colleagues showed that the overexpression in neurons of the amyloidogenic enzyme, BACE1, is sufficient to increase the risk of developing T2DM [80]. Therefore, this study demonstrates that neuronal BACE1 causes metabolic dysregulation throughout the body along with brain inflammation and cognitive impairment related to the process of amyloidosis. The PLB4 mouse presents a diabetic profile, thus demonstrating that neuronal BACE1 is in part responsible for the appearance of these peripheral metabolic alterations [84].

**Figure 2:**
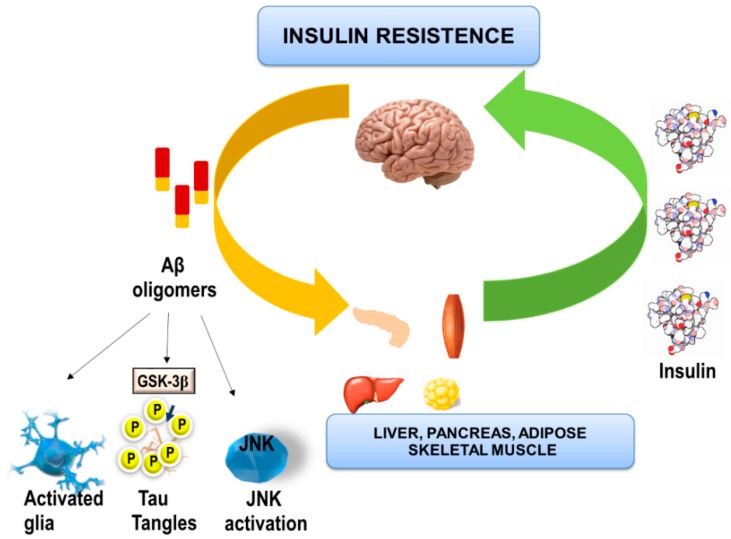
Aβ acting on the hypothalamus can dysregulate energy homeostasis in the human organism through a neuroinflammatory process. Furthermore, in the hippocampus, activation of glial reactivity could increase cytokine levels (such as TNF-α), activating c-Jun N-terminal Kinase and inducing IR resistance and TAU phosphorylation. Likewise, brain generated Aβ could accumulate in peripheral tissues such as the pancreas and skeletal muscle, favouring the appearance of T2DM.

Therefore, even though the hypothesis states that diabetic complications promote the onset and or progression of AD, the reverse scenario may also apply. This is also in agreement with the potential hypothesis that hyperglycaemia can also originate in the brain and affect the rest of the body (Figure 2). Meakin and colleagues demonstrated that knockout mice for BACE1^−/−^ are thin, resistant to obesity induced by high fat diet and show an increase in insulin sensitivity in peripheral tissues with a regulation of improved glucose metabolism throughout the body [85]. These results outline a novel aspect of BACE1 function in the regulation of metabolic homeostasis and, provide a possible connection between T2DM and AD [85]. 

## 8. Potential Pharmacological Approaches for Late Onset Alzheimer’s Disease Treatment Related with the Regulation of Insulin Metabolism

For all of the above, development of LOAD would pivot on the loss of IR functionality, oxidative stress and loss of control of protein homeostasis [86,87,88,89,90]. In order to modulate these mechanisms, different pharmacological approaches are proposed which may act in a combined and, potentially, synergistic manner. On the one hand, it may be appropriate to combine the use of antidiabetic drugs such as pioglitazone, intranasal insulin, NMDAR antagonists such as memantine and inhibitors of mTOR activity such as rapamycin and its derivatives (rapalogs) [22,44,91,92,93,94,95,96,97,98,99,100,101,102]. In all cases, they are drugs that have been validated in different Phase II (pioglitazone) and III (rapamycin) clinical trials. Since in no case did these molecules improve the evolution of patients with LOAD (in the different Phase III studies), this allows for the possibility of studying their possible synergies when administered in combination.

### 8.1. Antidiabetic Drugs. Modulators of Proliferation of Activated Gamma Peroxisome Receptor. Pioglitazone

Pioglitazone is an orally active antidiabetic drug in the family of thioazolidinediones, also called “insulin sensitizers” [93,94]. Pioglitazone is a potent and selective receptor agonist for the proliferation of activated gamma peroxisomes receptor (PPARγ). These receptors regulate the transcription of a number of genes that respond to insulin [93]. PPARγs are found in most tissues in which insulin exerts its action: adipose tissue, skeletal muscle and liver. Activation of these receptors regulates the transcription of genes involved in the control of glucose production, transport and its utilization. In relationship to LOAD, the treatment with pioglitazone has been shown to reduce glial pro-inflammatory activity and, to decrease Aβ peptide levels due to the phagocytic activity of microglia [92]. In 3xTg-AD mice treated with pioglitazone for 4 months, this drug improves brain spatial learning impairment, TAU hyperphosphorylation, and neuroinflammation [93]. In a recent preclinical study Fernandez-Martos and co-workers reported that the association of pioglitazone with leptin showed beneficial effects on the preclinical APPswe/PS1dE9 mice model of familial AD improving cognition and decreasing Aβ levels [102]. Recent studies indicate a very relevant effect of the drug reversing the damage that neuroinflammation causes in the structural plasticity of the dendrites. Thus, it has been observed that treatment with pioglitazone can reverse the loss of synaptic density induced by Aβ peptide generation [91]. Although preclinical data gives support to the potential beneficial effects of pioglitazone in AD, clinical data reported until now shows conflicting results regarding efficacy due to the many limitations of these trials [100,101]. Therefore, further clinical trials on the potential use of pioglitazone for the treatment of LOAD are necessary. Phase II clinical trials of the drug demonstrate that it is a safe and well tolerated molecule. Two Phase III trials are currently under way, of which conclusions regarding their effectiveness against AD cannot yet be obtained [91,100,101].

### 8.2. Intranasal Insulin for LOAD Treatment

In previous preclinical studies, intranasal treatments with insulin or insulin analogues have afforded some degree of memory improvement or of protection against cognitive deterioration in mice models of AD [101,102,103,104,105,106,107,108,109]. However, in a recent reported study (NCT01595646), Craft and co-workers reported that intranasal-administered insulin improves memory for adults with mild cognitive impairment and LOAD [108,109,110]. Furthermore, authors suggest that insulin could improve and modify the AD-related pathophysiologic processes. Another interesting point is that the therapeutic effects of insulin are modulated by APOE genotype. Accordingly, the study gives support to the continued investigation on potential stimulation of insulin receptor as a therapy for LOAD [108]. 

### 8.3. Rapalogs

It is well known that the PI3K/AKT/mTOR dysregulation may decrease the autophagic process leading to the accumulation of Aβ42 deposition and protein aggregation [44]. Likewise, mTOR is involved in the modulation of IRS1 activity, representing one of the best-characterized events leading to insulin resistance [44]. Therefore, alteration or better activation of the mTOR pathway could represent an important link between Aβ and insulin signalling, providing new insights into the relationship between insulin resistance and incidence of AD.

mTOR is a kinase involved in energy and protein homeostasis in cells. Both rapamycin and its derivatives prevent the formation of the mTORC1 complex, acting as allosteric inhibitors [109,110,111]. However, the main limitations of rapamycin are its solubility, long half-life and the poor oral absorption making it necessary for the development of analog molecules, among them temsirolimus, which is an ester derived from rapamycin, soluble in water and suitable for administration both oral and intravenously. Its use was approved in 1977 by the FDA and the European Medicines Agency for the treatment of renal carcinoma [111]. Both drugs have the same mechanism of action. Jiang and co-workers recently reported that temsirolimus promotes autophagic clearance of Aβ, exerts protective effects accompanied by an improvement in spatial cognitive functions in APP/PS1 model of familial AD [102]. This study give support to the therapeutic potentials of temsirolimus in preclinical models of AD.

## 9. Conclusions

Given the amount of data of which we are in possession now, it can be concluded that the pathology hereby described as LOAD is very closely related to the alterations derived of insulin resistance. Effective energy metabolism is the base on the proper functioning of cellular types and, when disrupted, affects negatively their function. In the case of neurons, which are having glucose as its main energy source, this situation can be utterly disastrous leading to their ineffective activity and consequently cognitive decline. That is why the IR has such a prominent role.

It is now well established Aβ could bind to the IR in the hippocampus, revealing important cognitive loss, when the receptor is inhibited and enhancing the neurodegenerative process in this brain region. Moreover, Aβ bind to the liver IR in the preclinical APPswe/PS1E9 mice model of familial AD, suggesting the possibility that decreases of Aβ production may be a novel potential treatment for use in T2DM (Figure 2) [73,74,112]. Lastly, the therapeutic potential of Aβ inhibitors (for example BACE 1 inhibitors) has not yet been verified in clinical trials. However, antidiabetic therapies such as pioglitazone or intranasal insulin are more likely to be effective in individuals with LOAD. Therefore, we suggest that the future timing of a more effective LOAD therapy should be the key factor in determining if T2DM drugs shown beneficial actions in LOAD. Targeting the early stages of LOAD, before widespread cognitive loss due to synapses and neurons degeneration has occurred is likely to produce the best clinical outcome, but identification of individuals at this stage of LOAD is difficult. Accordingly, the modulation of brain IR preventing its inactivation could be a suitable strategy in a combinatory strategy therapy for LOAD.

## Figures and Tables

**Table 1:** Examples of IR signalling pathway alterations in the brain in late onset Alzheimer’s disease.

Reference	Physiological Alterations	Pathological Effects
Biessels and Reagan, 2015 [36]	Down regulation in neurogenesis were associated with reductions in dendritic spine density in CA1 pyramidal neurons.	Learning and memory loss.
Hoyer, S., 2004 [26]	Decline in ATP levels (mitochondrial alteration). PKB activity inhibition GSK3 activity increase.	Amount in TAU phosphorylation. Oxidative stress increases
De Felice, F.G., 2014 [29]	Neuroinflammation and TNFα increase associated with neuronal ER stress and JNK activation	Brain IR down regulation and synaptic alteration.
Grillo, C.A., 2015 [42]	Hippocampal-specific insulin resistance using a lentiviral vector expressing an IR antisense sequence	Down regulation of GluN2B and GluA1 phosphorylation at synapses. Memory failure independent of peripheral metabolic alterations.
Hoyer, S., 1994 [28]	Insulin modulates levels of acetylcholine and norepinephrine neurotransmitters,	Cognition loss
Frolich, L.D., 1999 [25]	Formation and deposition of advanced glycation end products (AGEs)	Up-regulate APP via oxidative stress and Aβ production enhancement
De Felice and Ferreira, 2014 [30]	mTOR dysregulation	Learning and memory deficits, cell cycle reentry
Craft, S. 2012 [6]	Insulin resistance increases vascular dysfunction	Vascular dementia
Craft, S. 2005 [43]	Insulin resistance inhibits IDE activity	Aβ levels Increase
